# Robotic-Assisted Hysterectomy for Benign Indications of Uteri Less Than Fourteen Weeks Size Versus More Than Fourteen Weeks Size: A Comparative Study

**DOI:** 10.7759/cureus.15263

**Published:** 2021-05-26

**Authors:** Anupama Bahadur, Mamta Kumawat, Latika Chawla, Dhriti Kapur, Yogesh Bahurupi, Rajlaxmi Mundhra

**Affiliations:** 1 Obstetrics and Gynecology, All India Institute of Medical Sciences, Rishikesh, Rishikesh, IND; 2 Obstetrics and Gynecology, All India Institute of Medical Sciences, New Delhi, Delhi, IND; 3 Community and Family Medicine, All India Institute of Medical Sciences, Rishikesh, Rishikesh, IND

**Keywords:** robotic surgery, hysterectomy, da vinci surgical system, benign hysterectomy, gynaecology

## Abstract

Objectives

This study was conducted to evaluate the feasibility of robotic hysterectomy for benign indications in patients with small size (<14 weeks) versus large size (>14 weeks) uterus.

Methods

This prospective study was conducted in a single centre from August 2018 to January 2020 in the Department of Obstetrics and Gynecology at All India Institute of Medical Sciences, Rishikesh (Uttarakhand). Surgical outcomes of 216 patients who underwent a robotic hysterectomy in our institution for benign indications were analysed. Women opting for definitive surgical management by minimally invasive technique were divided into two groups according to the size of the uterus less than 14 weeks (group 1) versus more than equal to 14 weeks (group 2). Data collected in both groups included intra-operative and post-operative parameters, length of hospital stay and morbidity if any.

Results

The demographic profile was comparable in both groups. The mean estimated blood loss was 180.78 ±68.0 ml (range, 10-340 ml) in group 1 and 253.49 ±57ml (range, 60-360 ml) in group 2 (p-value < 0.0001). However, the fall in haemoglobin level after 24 hours of surgery was not statistically significantly different between the two groups. The total duration of surgery in group 1 was 97.86 ± 12.0 minutes (range, 78-132 minutes) and in group 2 was 116.60 ± 15.4 minutes (range, 97-156 minutes), the difference being statically significant (p-value < 0.0001, 95% CI 103±2.1). Console time in group 1 was 43.84 ±6.0 minutes (range, 34-57 minutes) and in group 2 53.22 ±5.5 minutes (range, 44-66 minutes), the difference being statistically significant (p-value < 0.0001, 95% CI 46.57±0.97). There was no difference observed in terms of intra-operative and post-operative complications between the two groups.

Conclusion

The total duration of surgery and estimated blood loss were directly proportional to the size of the uterus. However, complication rate, hospital stay and requirement of post-op analgesia were comparable in both groups. Robotic surgery in a larger uterus is a feasible option in terms of better surgical outcomes and postoperative course. Thus, robotic hysterectomy in women with a large uterus is a suitable approach in the narrow region of the pelvis.

## Introduction

Hysterectomy for benign diseases is the most common gynaecological surgery performed worldwide [1,2]. According to the National Family Health Survey (NFHS 4 survey 2015-16), in India, 6% of women in the age group of 30-49 years had undergone hysterectomy for heavy menstrual bleeding or chronic pelvic pain (56%), followed by fibroids (20%) [3].

More than 50% of hysterectomies are being performed via abdominal approach currently, but gradually the trend is shifting towards minimally invasive surgery as it offers the advantage of lesser blood loss, less post-op pain, early mobilization, faster recovery, shorter duration of hospital stay, and reduced patient morbidity [4,5]. In 2005, FDA approved the da Vinci Surgical System for robotic gynaecological surgeries and since then its use has increased in both benign and gynae-oncological surgeries. With the robotic approach gaining acceptance for hysterectomy, several difficulties faced during the laparoscopic approach have been overcome. The benefits of robotic surgery are parallel to those of laparoscopic surgery [6]. However, the advantages offered by robotic-assisted surgery include seven degrees of movement along with elimination of hand tremors, endo-wrist movements, flexible movements thereby eliminating fulcrum effect of laparoscopy, stable camera, clear surgical three-dimensional (3D) stereoscopic view of the surgical field and increased independence to operating surgeon with a shorter learning curve [7-9]. Surgeons’ performance is enhanced due to the ease and comfort of the robotic console. The surgeon performs the surgery with lesser fatigability and better ergonomics. The disadvantages include high cost and lack of tactile feedback while working in restricted operating fields such as the human pelvis [10-12].

Previously few studies analysed the impact of uterine size on surgical outcomes of hysterectomy by minimally invasive routes. O'Hanlan et al. analysed the impact of uterine size on outcomes of laparoscopic hysterectomy comparing uterine weights of <250 g to those >250 g [13]. Payne et al. studied the impact of uterine weight on surgical outcomes on 256 patients undergoing robotic hysterectomy based on the division of cases into two groups less than 500 g and more than 500 g and concluded that blood loss and operating time increased as the size of the uterus increased [14]. Akazawa et al. analysed the surgical outcome of 527 patients of robotic hysterectomy after separating them into five groups and observed an increase in operating time and blood loss as uterine size increased [15]. Tyan et al. reported robotic hysterectomy as a safe and feasible option for benign cases across a range of uterine weights [16].

There exists no linear association between uterine size and surgical outcome in the literature. There is no definite cut-off size based on which it can be said that surgical outcome gets better with minimally invasive surgery as compared to the open surgical approach. There is no prospective study from the Indian sub-continent that has compared the feasibility of performing robotic hysterectomy for small versus large size uteri in benign conditions. In this study, we compared robotic hysterectomy for benign conditions based on uterine size less than 14 weeks and more than 14 weeks. Below 12 weeks uterus remain a pelvic organ, and hence, we chose 14 weeks as the cut-off size to divide our cases. 

## Materials and methods

This was a single-institution study involving patients undergoing robotic hysterectomy for benign indications in the Department of Obstetrics and Gynaecology at All India Institute of Medical Sciences Rishikesh, Uttarakhand, India. We conducted this prospective study over a period of 18 months from August 2018 to January 2020. 

All surgically fit patients with benign indications were included as part of our study. We excluded those patients who had either suspected or confirmed malignancy and converted them to laparotomy. A single surgeon performed all robotic hysterectomies using the da Vinci Xi model. We divided the patients into two groups based on uterine size determined preoperatively by a bimanual pelvic examination done by the operating surgeon. Group 1 included patients with the size of the uterus less than 14 weeks and group 2 included those with uterus size more than and equal to 14 weeks size. Indications for hysterectomy were abnormal uterine bleeding of varying causes not responding to medical management, chronic pelvic pain refractory to medical therapy and severe endometriosis.

Baseline demographic profile including patient’s age, parity, body mass index (BMI), previous uterine surgeries were noted in a predesigned proforma. Various intra-operative parameters including docking time, console time, need for intraoperative adhesiolysis, time taken for vault suturing, total surgery time, the weight of the uterus, number of robotic arms used, number of assistant ports used, number of times instruments changed, number of time camera got smudged and required cleaning, any complications were noted. Post-operative parameters like the need for blood transfusion, fall in haemoglobin, post-operative analgesia requirement, duration of hospital stay (the day of surgery to the day of discharge), major and minor complications were noted and analysed. The total duration of surgery was calculated from the first time skin incision was given till skin closure. We calculated estimated blood loss by subtracting irrigation fluid from suction jar fluid. Both the groups were compared based on baseline characteristics and surgical outcomes both intraoperative and postoperative.

Statistical analysis was performed using “SPSS version 24” (IBM Corp., Armonk, NY). Descriptive statistics such as mean, median and standard deviation were calculated. An independent t-test was applied to compare the mean of continuous variables following a normal distribution. We applied non-parametric tests like Mann Whitney U test where continuous variables did not follow a normal distribution. To compare categorical variables, the Chi-squared test and Fisher’s exact test were applied. To indicate statistical significance, a p-value < 0.05 was used. Ethical clearance was obtained from Institutional Ethics Committee vide no 243/IEC/PGM/2018.

## Results

**Figure 1:**
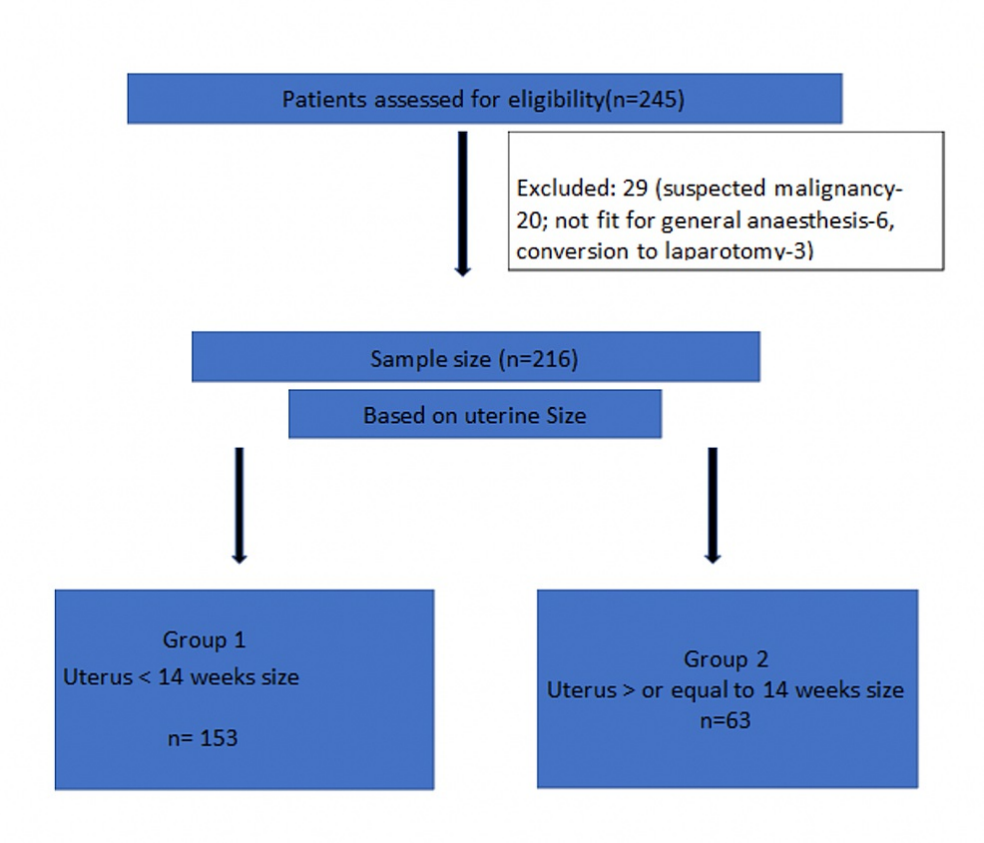
Patient flow diagram

During the specified time period, we assessed 245 patients for eligibility. However, based on inclusion and exclusion criteria, 216 patients were finally recruited. Group 1 included 153 patients and group 2 included 63 patients based on the size of the uterus, less than 14 weeks and more than 14 weeks, respectively (Figure 1).

Baseline characteristics

Both the groups had similar baseline characteristics as shown in Table 1. The mean age in group 1 was 44.28 ± 4.78 years (range, 35-55 years) and 44.26±4.60 years (range, 36-55 years) in group 2. Mean BMI was 25.94±3 kg/m^2^ (range, 19-38.3 kg/m^2^) in group 1 and 26.50±4.6 kg/m^2^ (range, 20.2-43.2 kg/m^2^) in group 2. Mean parity was 3.03±1.3 in group 1 and 2.80±1.1 in group 2. In group 1, nine patients out of 153 (5.8%) had a history of previous uterine surgery and three patients out of 63 (4.76%) in group 2 had a history of previous uterine surgery.

**Table 1:** Baseline characteristics *Meanׅ±standard deviation. Normality of distribution of variables checked using Shapiro Wilk test.

Variables	Group 1 (uterus < 14 weeks size; n=153)	Group 2 (uterus size ≥ 14 weeks; n=63)	p-Value
Age* (years)	44.28 ± 4.78	44.26 ± 4.60	0.9
Parity*	3.03 ± 1.3	2.80 ± 1.1	0.2
Body mass index* (kg/m^2^)	25.94 ± 3.9	26.50 ± 4.6	0.3
Previous uterine surgeries	9 (5.88%)	3 (4.76%)	0.98

As seen in Table 2, in group 1, 62 (40.52%) patients had AUB-L, 46 (30.06 %) patients had AUB-A, 11 (7.18%) patient had AUB-P, 20 (13.07%) patients had chronic pelvic pain and 14 (9.15%) patients had endometriosis. In group 2, 17 (26.98%) patients had AUB-L, 34 (53.96%) patients had AUB-A, 3 (4.76%) patient had AUB-P, 5 (7.90%) patients had chronic pelvic pain and 4 (6.34%) had endometriosis.

**Table 2:** Indications of hysterectomy AUB: abnormal uterine bleeding, L: leiomyoma, A: adenomyosis, P: polyp.

Indication	Group 1 (uterus < 14 weeks size; n=153)	Group 2 (uterus size ≥ 14 weeks; n=63)
AUB-L	62 (40.52%)	17 (26.98%)
AUB-A	46 (30.06%)	34 (53.96%)
AUB-P	11 (7.18%)	3 (4.76%)
Chronic pelvic pain	20 (13.07%)	5 (7.90%)
Endometriosis	14 (9.15%)	4 (6.34%)

**Figure 2:**
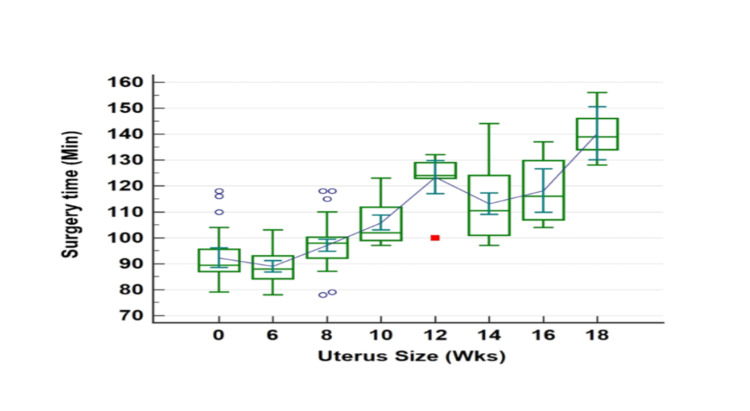
Relationship between surgery time and uterus size

Surgical outcomes like total duration of surgery, console time and estimated blood loss are described in Table 3. Total operating time in group 1 was 97.86 ± 12.0 minutes (range, 78-132 minutes) and in group 2 was 116.60 ± 15.4 minutes (97-156 minutes), the difference being statistically significant (p-value < 0.0001, 95% CI 103±2.1). As seen in Figure 2, surgery time increased with an increase in uterine size. Console time in group 1 was 43.84 ± 6.0 minutes (range, 34-57 minutes) and in group 2 was 53.22 ± 5.5 minutes (range, 44-66 minutes), the difference being statistically significant (p-value <0.0001, 95% CI 46.57±0.97). Docking time was 9.62 ± 2.4 minutes (range, 5-12 minutes) in group 1 and 8.92 ± 2.8 minutes (range, 4-16 minutes) in group 2, and this difference was not statistically significant (p-value < 0.06, 95% CI 9.41±0.34).

**Table 3:** Intraoperative parameters *Statistically significant.

Variables	Group 1 (uterus < 14 weeks size; n=153), mean ± SD (range)	Group 2 (uterus size ≥ 14 weeks; n=63), mean ± SD (range)	p-Value
Docking time (minute)	9.62 ± 2.4 (5-12)	8.92 ± 2.8 (4-16)	0.06
Total operating time (minute)	97.86 ± 12 (78-132)	116.6 ± 15.4 (97-156)	0.0001*
Console time (minute)	43.84 ± 6 (34-57)	53.22 ± 5.5 (44-66)	0.0001*
Weight of uterus (g)	174.15 ± 61.9	346.12 ± 157.5	0.0001*
Vault closure (minute)	8.73 ± 2.6 (5-17)	8.03 ± 2.2 (5-15)	0.06
Specimen extraction time (minute)	50.70 ± 13.64 (21-83)	61.43 ± 15.78 (30-95)	0.001*
No. of Robotic arm used	3.01 ± 0.1	3.11 ± 0.3	0.0003*
Number of assistant ports used	1.05 ± 0.2	1.06 ± 0.2	0.73
Number of times instrument changed	1.33 ± 0.5	1.38 ± 0.5	0.5
No times camera got smudged and required cleaning	2.60 ± 0.9	2.61 ± 0.9	0.94

**Figure 3:**
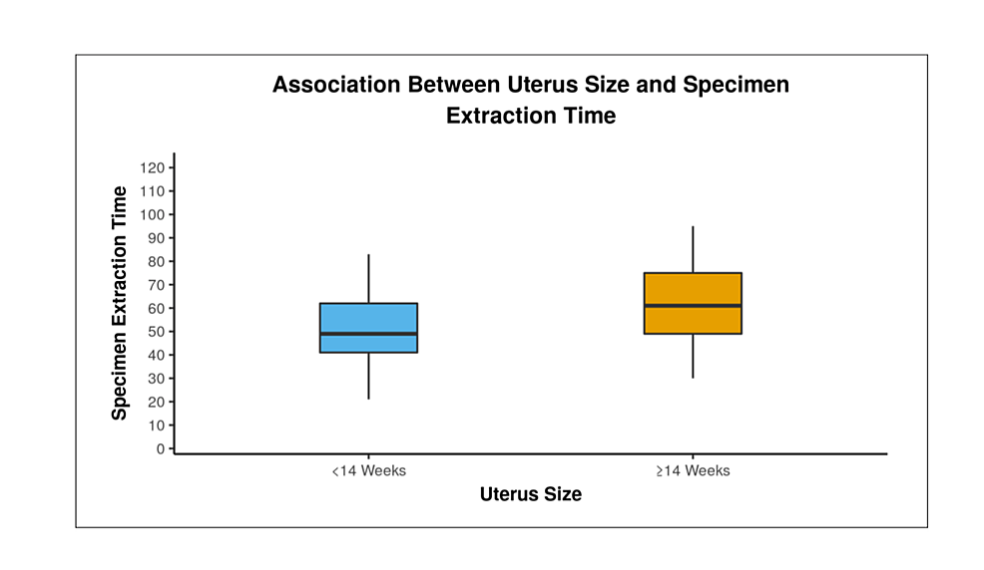
Comparison of specimen extraction time between two groups

Vault closure time in group 1 was 8.73±2.6 minutes (range 5-17 minutes) and in group 2 was 8.03±2.2 minutes (range, 5-15 minutes), however, the difference was not statistically significant between the two groups (p-value < 0.06, 95% CI 8.52±0.34). The time taken for docking and vault closure were initially high during the learning curve but as the surgeon acquired proficiency in technique the time taken was reduced and eventually reached a plateau. Specimen extraction time in group 1 was 50.70 ± 13.64 minutes (range, 21-83 minutes) and in group 2 was 61.43 ± 15.78 minutes (range, 30-95 minutes), the difference being statistically significant (p-value < 0.0001). Figure 3 shows increasing specimen extraction time with an increase in uterine size.

**Figure 4:**
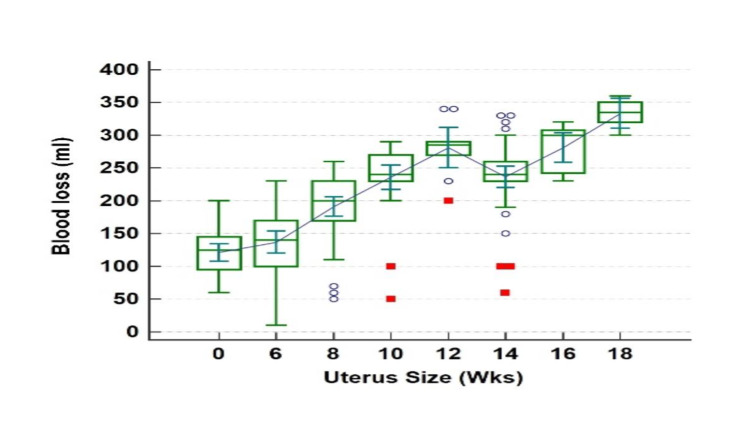
Relationship between blood loss and uterus size

In our study, the mean estimated blood loss (EBL) was 180.78 ±68.0 ml (range, 10-340 ml) in group 1 and 253.49 ± 57.6 ml (range, 60-360 ml) in group 2 (p-value < 0.0001). However, the fall in haemoglobin level after 24 hours of surgery was not statistically significantly different between the two groups. As seen in Figure 4, blood loss increased with increased uterine size.

Robotic surgery was done using two robotic arms (fenestrated bipolar and monopolar scissor) in the majority of cases. In group 1 two patients and in group 2 seven patients required the use of a third robotic arm (p-value < 0.0003) either Prograsp or Tenaculum for bowel retraction or manipulation of large uteri. Other parameters like extra assistant ports, camera cleaning, number of time instruments changed during surgery, need for intra-op adhesiolysis, requirement of blood transfusion were not significantly different between the two groups.

Post-Operative Outcome

Requirement of post-op analgesia was defined as analgesia used over and above usual practice in our department which was injection diclofenac 50 mg IV, eight hourly and injection Paracetamol 1 g IV six hourly for initial two postoperative days. In group 1 six patients (3.92%) and in group 2 three patients (4.76%) required post-operative analgesia which was not statistically significant between the two groups (p-value=0.72). In group 1, the mean duration of stay in hospital was 2.45±1.1 day (range, 1-4 days) and in group 2 was 2.46 ±1.0 days (range, 1-4 days; p-value =0.95).

Complication rate

Nine patients had either major or minor complications in our study. Major complications were bladder injury (one in each group), one patient had post-operative pulmonary edema which was managed in ICU and one patient had a ureterovaginal fistula which was dealt with by the urologist. Minor complications were discharge per vaginum in two patients in group 1 which required admission and IV antibiotics. We readmitted two patients in group 1 and one in group 2 with fever. On further evaluation and workup for fever, it was observed that the cause of fever was typhoid in one patient, scrub typhus in another and urinary tract infection in the third patient. No surgical site infection or vault dehiscence were observed in our study. Complication rates in either group were not statistically significant.

## Discussion

With the advent of robotic surgery, the clinical scenario has changed for the operating surgeon. Difficulties encountered during laparoscopic surgeries in large uterine sizes like limited access to the vascular pedicle and restricted uterine manoeuvrability are better handled with a robotic approach. With the addition of endo-wrist technology in robotics, complex surgeries can be easily performed making them the same as open surgical manoeuvres. Moreover, the operating surgeon has the camera in his/her control with a stable surgical field as compared to laparoscopy.

In this study, we report the outcome of benign robotic hysterectomy in a larger uterus as compared to a small-sized uterus. Though surgery for a large uterus was more time-consuming and associated with increased blood loss, other parameters like docking time, vault closure time, the requirement of post-op analgesia, hospital stay and complication rates were similar in both groups. Blood loss during robotic hysterectomy was higher in group 2 (p-value of < 0.0001) and the difference was statistically significant. Console time and total operating time were higher in group 2 as compared to group 1 and the difference was statistically significant. This was due to increased manipulation and time taken in group 2 owing to larger uteri. In our study, there was a correlation between uterine size and total time of surgery, as the size of the uterus increased the total time taken for surgery increased which was statistically significant (p-value < 0.0001). Robotic hysterectomy in women with a large uterus is a suitable approach in the narrow region of the pelvis.

Akazawa et al. in 2019, conducted a retrospective study on the impact of uterine weight and its outcome in robotic hysterectomy. They analysed the surgical outcomes in 527 patients who had a robotic hysterectomy performed for benign indications. Patients were divided into five groups based on uterine size: <250 g, 250-500 g, 500-750 g, 750-1000 g, and >1000 g. Estimated blood loss and operating time showed a linear relationship with uterine size (p-value<0.001). The blood loss during surgery was more in a group with uterus weight >1000 g [15]. Similar to the study by Akazawa et al., we also found that mean blood loss and operating time increased as the size of the uterus increased. In a similar study by Silasi et al. in 2013, the authors compared robotic hysterectomy with laparotomy for very large uteri (defined as uterus weighing more than 1000 g). The authors observed that the median blood loss was 150 ml (range, 50-700 ml) in patients who underwent robotic surgery as compared to 425 ml (range, 50-1000 ml) for open surgery (p<0.001). The total operative time in robotic surgery was 225 minutes and 150 minutes in laparotomy. The authors concluded that robotic surgery is a feasible option in patients with very large myomatous uteri as it has minimal morbidity [17].

Console time was defined as the time taken from the end of docking to complete colpotomy, and in our study, we observed that it was statistically significantly higher in group 2 as compared to group 1. We did not include vault closure time in console time. This was due to the fact that specimen extraction time varied for all cases. Mean console time in group 1 was 43.84 ± 6.0 minutes, ranging from 34 to 57 minutes. In group 2, the mean console time was 53.22 ± 5.5 minutes, ranging from 44 to 66 minutes (p-value < 0.0001).

The third robotic arm was used in 1.3% of patients in group 1 and 11.11% of patients in group 2 (p<0.0003) which indicates that there is a correlation between uterus size and the number of robotic arms used. In group 2, the use of the third arm of the robot was most frequently required for either bowel retraction or manipulation of the large uteri using Prograsp or Tenaculum. No correlation between uterus size and docking or vault closure time was observed in our study. In the initial cases, docking and vault closure time was higher but as the surgeon gained proficiency in this technique the time taken decreased progressively and become consistent. Also a day prior to surgery, support staff and other faculties (anesthesiologists and nursing staff) were made aware of the surgery and the critical steps involved were discussed. This helped in curtailing the learning curve of the team.

In our study, totally nine patients had intra-operative and post-operative complications and this was similar in both groups (p < 0.48). Akazawa et al. in 2019 studied the outcome of robotic hysterectomy in 527 patients who had 18 cases (3.4%) with postoperative complications [15]. Petersen et al. in 2018 studied urologic injury with a robotic hysterectomy and observed urologic injury in 0.92% of cases [18].

The main limitation of this study was being a single centre study and the number of cases were less. We, therefore, require multicenter trials with a larger sample size to further evaluate the feasibility of robotic hysterectomy in larger size uteri.

## Conclusions

From the results of our study, we can conclude that there is no difficulty in performing robotic hysterectomy for larger uteri. Robotic hysterectomy may be a feasible option for patients with uterine size more than or equal to 14 weeks size. With a small incision, better cosmesis, better clinical outcome and patient satisfaction are achieved with this novel technique. As surgeons, we need to master not only the nuances this technique has to offer but also master the machine. We must remember that robotics is nothing but laparoscopy with a computer interface.
